# How Does the Extraction of Remaining Lipids From Babassu Press Cake Using Green and Traditional Solvents Affect the Oil and Defatted Solids?

**DOI:** 10.1111/1750-3841.71275

**Published:** 2026-07-07

**Authors:** Clara Santa Rosa Fioriti, Paloma Jamily Cristina Magalhães, Paola de Cássia Franco Visioli, Keila Kazue Aracava, José Pedro Zanetti Prado, Christianne Elisabete da Costa Rodrigues

**Affiliations:** ^1^ Laboratório de Engenharia de Separações (LES), Departamento de Engenharia de Alimentos (ZEA), Faculdade de Zootecnia e Engenharia de Alimentos (FZEA) Universidade de São Paulo (USP) Pirassununga São Paulo Brazil

**Keywords:** babaçu, ethanol, *Orbignya phalerata*, physical properties, protein solubility, solvent extraction

## Abstract

Babassu (*Orbignya phalerata*) plays a crucial role in several indigenous and quilombo communities in the north and northeast regions of Brazil. Mechanical pressing of nuts yields babassu oil of interest to the cosmetic and pharmaceutical industries. However, the solid fraction from pressing (babassu press cake, BPC) has rarely been explored, thereby devaluing its potential for application in the food industry. Therefore, this study comprehensively characterized BPC, extracted residual lipids with hexane and ethanol, and characterized the extracted babassu oils (BO.H and BO.E) and the corresponding defatted solids (DS.H and DS.E). Ethanol resulted in a high oil extraction yield (85.8 ± 0.5%) and a low liquid holdup (0.57 ± 0.03 kg adhered solution/kg of fiber) at 75°C, in addition to a reduction in the DS.E's aflatoxin values compared with those of BPC. BO.E's composition in fatty acids and triacylglycerols and physical properties such as density and viscosity were similar to those of BO.H. BPC, DS.H, and DS.E had low nitrogen solubility indices (NSIs of 14.3 ± 0.8%, 11.27 ± 0.09%, and 9.9 ± 0.3%, respectively), even at alkaline pH values, indicating that protein recovery from BPC is challenging. Water (WAC) and oil (OAC) absorption capacities are similar for BPC, DS.H, and DS.E, indicating that the lipid extraction process has an insignificant effect on these properties. The results reveal the potential of incorporating residual oil and defatted solids from BPC in various applications, contributing to the valorization of the entire babassu production chain.

## 1 Introduction

Press cakes are agroindustrial byproducts obtained from the mechanical pressing of oilseeds with high lipid content, including both conventional sources, such as canola, sunflower, and peanut (Vidal et al. 2022; USDA 2025), and unconventional oilseeds from the Amazon biome (Porro and Sousa 2023). These materials, traditionally used as animal feed or for energy generation, are rich in proteins, lipids, fibers, and minor compounds with potential applications in human nutrition, which has increased interest in their valorization (Nevara et al. 2023; Silva et al. 2023).

In the Amazon context, babassu (*Orbignya phalerata*) stands out for its high lipid content (50%–67%) in its nuts, which also contain significant amounts of proteins (8%–12%) and carbohydrates (17%–27%) (Ferreira et al. 2023). Babassu is the second most important nontimber forest product in Brazil. In 2024, babassu nut production totaled 25,572 tons, generating revenue of USD 15,200, with Maranhão state as the leading producer (IBGE 2026).

The high oil content of babassu nuts indicates that they are processed by mechanical pressing to obtain babassu oil, which is rich in lauric acid (40%–55%) and is used mainly in cleaning and hygiene products and in the cosmetic and pharmaceutical industries as an ingredient of creams, lubricants, and soaps (Bauer et al. 2020; Codex Alimentarius Commission 1999). In addition to oil, the mechanical pressing of nuts generates a solid fraction known as press cake, which still contains a specific lipid content (4.5%–12.2%) (Sampaio Neto et al. 2020; Ferreira et al. 2023). However, the use of this cake has rarely been explored, thereby devaluing its potential application in the food industry (Bauer et al. 2020).

Studies have focused on the application of babassu press cake (BPC) in animal feed (Oliveira et al. 2021), biofuel production (Silva et al. 2023), fermentation processes (de Castro et al. 2016), and residual lipid extraction (Sampaio Neto et al. 2020).

Because BPC has a low lipid content, residual lipids must be recovered by solvent extraction (Kemper 2005). The solvent traditionally used for oil extraction from oleaginous matrices is hexane, a mixture of *n*‐hexane isomers. The main advantage of this solvent is its complete miscibility with triacylglycerols (TAGs), even at room temperature, which results in high extraction yields and, consequently, defatted meal with low lipid content, as well as high stability and low corrosiveness to equipment. However, hexane has significant disadvantages, including high flammability, risks to human health due to its toxicity, fossil origin, and environmental impacts (Cravotto et al. 2022).

Several solvents, such as short‐chain alcohols (ethanol and isopropanol), terpenes, cyclopentyl methyl ether (CPME), 2‐methyltetrahydrofuran (2‐MeTHF, also known as 2‐methyloxolane) (Claux et al. 2021), eutectic solvents, supercritical CO_2_, and ionic liquids (de Jesus and Filho 2020; Gasparetto et al. 2022), have been suggested as potential substitutes for hexane. Among those suggested, ethanol stands out for its significant advantages and can serve as an alternative solvent for oil extraction. This solvent is derived from renewable sources, such as plants and roots, including sugarcane, corn, and beetroot (Bessa et al. 2017; Toda et al. 2023). From an environmental perspective, ethanol has significant advantages and is classified as a Class 3 substance by the Food and Drug Administration (FDA 2017). Compared with hexane, ethanol stands out for its lower flammability, lower environmental impact, and lower risk to human health, with Brazil being the second‐largest producer in the world (RFA 2025).

Ethanol, due to its higher polarity compared to hexane, exhibits lower selectivity and solubility for TAGs. However, these limitations can be mitigated by adjusting the extraction process configuration, such as increasing the solvent‐to‐solid ratio, raising the temperature, and increasing the number of stages of contact between solid and solvent. As noted by Magalhães et al. (2023), to achieve a cumulative extraction yield statistically equivalent to that of hexane in the extraction of peanut press cake oil, three extraction stages with ethanol were required at 75°C using a solvent/solid mass ratio of 5, whereas hexane required only two stages at 55°C and a lower amount of solvent (solvent:solid ratio of 4).

Studies have examined the feasibility of using ethanol as a solvent for the recovery of residual lipids from press cakes, such as peanut cake (Magalhães et al. 2023), walnut cake (Subra‐Paternault et al. 2022), and baru nut cake (Aracava et al. 2022). These studies indicate that ethanolic extraction yields high‐quality oil. However, the nitrogen solubility of the protein fraction contained in the defatted solid (DS) is decreased by ethanol, indicating that this more polar solvent can induce protein denaturation (Magalhães et al. 2023; Aracava et al. 2022).

With respect to the BPC, Sampaio Neto et al. (2020) evaluated the feasibility of using ethanol to extract oil from the press cake, performing a study on liquid‒liquid and solid‒liquid equilibria. Regarding liquid‒liquid equilibrium, the authors studied the solubility of babassu oil in anhydrous ethanol and reported that complete miscibility between the oil and the renewable solvent is achieved at temperatures above 35°C. With respect to oil extraction from BPC, the authors conducted solid‒liquid equilibrium trials evaluating the parameters of the solid‒liquid (BPC)‐to‐solvent mass ratio (1:20–3:5) and temperature (25°C, 35°C, and 45°C), with a contact time of 48 h. Although the liquid‒liquid equilibrium study revealed that ethanol solubilizes babassu oil, the solid‒liquid equilibrium revealed a strong interaction between the oil phase and the solid phase, resulting in oil extraction yield values ranging from 57% to 75%, regardless of the solid‒solvent ratio and temperature used. These results indicate the need for additional studies on extracting residual babassu oil from BPC using ethanol, including higher temperatures and expanding the characterization spectrum to enable inference on the need for refining. Moreover, to the best of our knowledge, no study has evaluated the characteristics of DSs obtained from the solvent extraction of babassu oil. These DSs may contain high levels of protein and fiber and could be used to recover valuable nutritional compounds.

Therefore, the aim of this study was to extract residual lipids from BPC using the conventional solvent hexane and the renewable alternative substitute ethanol. BPC, the oils obtained with hexane and ethanol, and the DSs resulting from solvent extraction were evaluated through physical and chemical characterization, with the aim of proposing industrial applications and promoting the valorization of the babassu coconut production chain.

## 2 Materials and Methods

### 2.1 Materials

BPC was supplied by COPPALJ (Cooperativa de Pequenos Produtores Agroextrativistas do Lago do Junco, Maranhão, Brazil; 4°35ʹ45.2ʺS, 45°02ʹ39.6ʺW). A batch obtained in October 2023 was refrigerated until use. Reagents included *n*‐hexane and absolute ethanol (≥98%, Synth, Brazil), β‐carotene (≥98%, Ambeed, USA), and d‐(+)‐glucose (≥98%, Sigma–Aldrich, USA).

### 2.2 Methods

#### 2.2.1 BPC Characterization

Before extraction, BPC was milled (Catel TE680, Brazil), and the 14–20 mesh fraction was selected for the trials. Particle diameter was calculated following ASAE (1995). True density (*d*_T_, kg/m^3^) was measured by helium pycnometry (Quantachrome MVP‐6DC, USA), while apparent density (*d*_A_, kg/m^3^) and bed porosity (*ε*, %) were determined as described by Magalhães et al. (2023).

BPC was analyzed for moisture (AOCS Ac 2–41, 2009; Nova Orgânica N035/3, Brazil), lipids (AOCS Am 5‐04, 2009; Ankom XT10, USA), ash (AOAC 2007; Marconi MA385/3, Brazil), starch (AOAC 996.11, 2007; AACC 76‐13.01, 2000; Megazyme Total Starch Kit), and total nitrogen (AOCS Ba 4f‐00, 2009; Leco FP‐528, USA). Total nitrogen was converted to protein using a factor of 5.3, as recommended by the AOAC (2007) for coconut and other tree nuts.

Soluble and insoluble fibers in BPC were determined enzymatically (Asp et al. 1983), while soluble carbohydrates were measured by the phenol–sulfuric acid method (Perkin Elmer Lambda 35, USA) at 490 nm using a glucose standard curve (DuBois et al. 1956). Nonfibrous carbohydrates were calculated by difference.

The contents of phosphorus (P), sulfur (S), calcium (Ca), magnesium (Mg), potassium (K), copper (Cu), iron (Fe), manganese (Mn), and zinc (Zn) were determined on the BPC according to the methodology proposed by Nogueira and Souza (2005). The dried and defatted sample was subjected to wet digestion with a nitro‐perchloric solution (65% HNO_3_ + 72% HClO_4_, 2:1 v/v, Synth, SP, Brazil). P and S were determined by spectrophotometry (Femto 600 S, Brazil) at wavelengths of 660 and 420 nm, respectively. The contents of Ca, Mg, Cu, Fe, Mn, and Zn were quantified using an atomic absorption spectrometer (Varian, Fast Sequential AA240FS, USA), with absorbance readings performed at element‐specific wavelengths (231.9 nm for Zn, 248.3 nm for Fe, 279.5 nm for Mn, 285.2 nm for Mg, 324.8 nm for Cu, and 422.7 nm for Ca). Potassium content was determined using a flame photometer (Micronal B462, Brazil), at wavelengths of 766 and 767 nm. All standards used to construct the calibration curves were supplied by Sigma–Aldrich (Bellefonte, PA, USA).

Aflatoxins (B1, B2, G1, and G2) were analyzed by liquid chromatography–mass spectrometry following the method of Sulyok et al. (2007), with modifications described in Franco et al. (2018). Briefly, a Waters Acquity I‐Class Ultra‐Performance LC system (Waters, Milford, MA, USA) equipped with a BEH C18 column (2.1 × 50 mm, 1.7 µm) and coupled to a Xevo TQ‐S mass spectrometer (Waters, Milford, MA, USA) was used. The mass spectrometer was operated in MRM mode using electrospray ionization in positive and negative ion modes. Data collection and processing were performed using software MassLynx version 4.1.

BPC color was measured using a noncontact reflectance spectrophotometer (HunterLab, USA), recording L*, a*, and b* values. Hue angle (°hue) was calculated following the method of McGuire (1992).

The solubility of the BPC protein fraction, commonly expressed as the nitrogen solubility index (NSI, %), was determined according to the methodology of Morr et al. (1985) (Equation 1), considering water as the solvent, at the following pH values: 2, 4.5, 6, and 9, adjusted using 1 N NaOH and 1 N HCl, at 25.0°C ± 0.1°C, with stirring at 400 rpm for 2 h, and using different solid‒water mass ratios (1:15, 1:25, 1:35, and 1:50). A Pyrex jacketed cell coupled to a thermostatic bath (Marconi, MA184, Brazil) was used to control the temperature. The nitrogen content of the extract was quantified by the Kjeldahl method (AOAC 2007), with modifications. For the liquid samples, 3 g was used to determine nitrogen content after 24 h of predigestion. Digestion was carried out at 400°C, with no heating ramp. The NaOH concentration was increased to 50% by adding 15 mL of the solution during distillation, and the dyes used to change color were methyl red and methylene blue.

(1) $$\mathrm{NSI}\left(\%\right)=\frac{N^{\mathrm{liquid}\;\mathrm{phase}}\;M^{\mathrm{liquid}\;\mathrm{phase}}}{N^{\mathrm{BPC}}\;M^{\mathrm{BPC}}}\times 100,$$

where *N* ^liquid phase^ and *N* ^BPC^ are the nitrogen contents (%) in the filtrate and BPC, respectively, and *M* ^liquid phase^ and *M* ^BPC^ are the water and BPC masses (g), respectively.

BPC was also characterized according to water absorption capacity and oil absorption capacity (WAC and OAC, respectively) according to the methodology suggested by Aracava et al. (2022).

All characterization analyses were performed at least in triplicate, and an experimental setup is shown in Figure S1.

#### 2.2.2 BPC Residual Oil Recovery Using Solvent Extraction

BPC was subjected to solvent extraction using *n*‐hexane (H) and ethanol (E) to recover residual lipids. Ethanol extraction was optimized for temperature based on oil yield and protein solubility of the DSs. Recovered oils were fully characterized and compared.

A stainless‐steel isothermal cylindrical batch extractor (500 mL; Tecnal TE‐139‐E4, Brazil; Marconi MA‐483/EC2, Brazil) was used for all oil extractions. The extractor was not pressurized but was hermetically sealed to prevent solvent loss. The extractor has several key components: a manometer, a mechanical agitator, a temperature controller, a bottom valve for withdrawing the extract phase, and a basket for packing the BPC. This basket is constructed from perforated stainless steel, is resistant to organic solvents and temperature, is not restrictive to the flow of extract and solvent, and allows extraction without solid particles (R. Oliveira et al. 2012). The BPC and solvent (hexane or ethanol) were weighed for all extraction experiments using a precision analytical balance (0.0001 g, Adam, PW‐254, Milton Keynes, UK), while adhering to the solid‐to‐solvent mass ratio.

Oil extractions were performed in single or multiple stages using a cross‐flow setup (Toda et al. 2022), in which partially defatted BPC serves as feed to subsequent stages with fresh solvent until the target yield is reached (Figure S2). The agitation parameter was maintained at 200 rpm for both sets of trials. Each extraction experiment was performed in duplicate.

Considering that two output streams, the liquid stream (extract phase) and the solid stream (DS), are obtained from each extraction stage, these phases were desolventized and characterized as detailed in Sections 2.2.3 and 2.2.4.

##### 2.2.2.1 BPC Residual Oil Extraction via Hexane

Oil extraction trials using hexane as a solvent were performed only for comparison with oil extracted using ethanol. The extraction conditions were selected for analytical purposes at 25°C, with a solid‐to‐solvent mass ratio of 1:4 over three extraction stages, each lasting 1 h (Figure S2). After extraction, the extracts (*n*‐hexane + oil) from each stage were pooled and rotary‐evaporated to recover the oil (Heidolph Hei‐VAP Silver, Germany) at 50°C under decreasing pressure (30–10 kPa). The oil from rotary evaporation was rested in a fume hood until constant mass, yielding babassu oil from hexane extraction (BO.H) for further characterization (Section 2.2.4).

Defatted solids from hexane extraction (DS.H) were also desolventized in a fume hood to constant mass and reserved for characterization (Section 2.2.3).

##### 2.2.2.2 BPC Residual Oil Extraction via Ethanol

BPC was extracted with absolute ethanol, a renewable green solvent, to obtain babassu residual oil (BO.E) and DSs (DS.E) for characterization.

Based on previous studies showing that ethanol and extraction temperature affect protein solubility (Magalhães et al. 2023), three temperatures (60°C, 75°C, and 90°C) were tested to optimize oil yield and nitrogen solubility (Section 2.2.3). These extraction trials were conducted in a single stage, using a solid‐to‐solvent mass ratio of 1:5 and an extraction time of 1 h.

The extract (ethanol + oil) obtained from extraction trials at the optimal temperature was rotary‐evaporated to recover the oil (Heidolph, model Hei‐VAP Silver, Germany) at 50°C with decreasing pressure from 10 to 5 kPa. The oil from rotary evaporation was subsequently placed in a vacuum oven (Tecnal, TE395, Piracicaba, São Paulo, Brazil) at 50°C until a constant mass was reached to ensure total solvent evaporation, allowing the generation of babassu oil from ethanol extraction (BO.E) for further characterization, as described in Section 2.2.4.

DS.E was desolventized in a forced convective oven (Nova Orgânica, N035/3, Piracicaba, São Paulo, Brazil) at 50°C for 48 h and reserved for characterization, as described in Section 2.2.3.

#### 2.2.3 Characterization of Defatted Solids From Oil Extractions via Hexane and Ethanol

Both DSs from hexane and ethanol extractions (DS.H and DS.E) were characterized for moisture, lipid, ash, and total nitrogen content (Section 2.2.1). DS.E was also evaluated for aflatoxins (B1, B2, G1, and G2) and soluble carbohydrates as described in Section 2.2.1.

Solvent performance was evaluated based on residual oil (RO, %) in the DSs, determined by the official method (AOCS Am 5‐04, 2009; Section 2.2.1). Oil extraction yield (*Y*_o__i__l_, %) was calculated from the lipid contents of BPC and DS (Equation 2).

(2) $$Y_{\mathrm{oil}}\left(\%\right)=\frac{M^{\mathrm{BPC}}\;w_{\mathrm{oil}}^{\mathrm{BPC}}-\;M^{\mathrm{DS}}\;w_{\mathrm{oil}}^{\mathrm{DS}}}{M^{\mathrm{BPC}}\;w_{\mathrm{oil}}^{\mathrm{BPC}}}\times 100,$$

where *M* ^BPC^ represents the mass of the raw material, $w_{\mathrm{oil}}^{\mathrm{BPC}}$ denotes the oil mass fraction in the raw material, *M* ^DS^ indicates the DS mass, and $w_{\mathrm{oil}}^{\mathrm{DS}}$ represents the residual oil mass fraction in the DS from each extraction stage.

Liquid holdup (LH; kg solution/kg DS) was determined by weighing solids before and after extraction using a semi‐analytical balance (0.01 g; Adam PGW‐1502i, UK).

To evaluate the effect of oil extraction on the DS instrumental color, the total color variation (ΔColor) was calculated with BPC as a reference (Equation 3).

(3) $$\Delta \mathrm{Color}=\sqrt{{\left(L^{*}-{L^{*}}_{\mathrm{BPC}}\right)}^{2}{\left(b^{*}-{b^{*}}_{\mathrm{BPC}}\right)}^{2}{\left(a^{*}-{a^{*}}_{\mathrm{BPC}}\right)}^{2}}$$

As described in Section 2.2.2, ethanol extraction was tested at 60°C, 75°C, and 90°C to optimize oil yield (Equation 2) and NSI (Equation 1). NSI of DSs was determined as in Section 2.2.1 using a 1:15 solid‐to‐solvent ratio; for DS.H, NSI was evaluated only at pH 9 for comparison.

#### 2.2.4 Characterization of Oils From Extractions via Hexane and Ethanol

Babassu oils from hexane and ethanol extractions (BO.H and BO.E) were subjected to methylation (AOCS Ce 2‐66, 2009) and analyzed for fatty acid composition by gas chromatography as fatty acid methyl esters (FAMEs) (Ce 1‐62, 2009). Analyses were performed using a Shimadzu 2010 AF capillary gas chromatograph (Japan), with an automatic injector (Shimadzu, model AOC‐20i, Japan) and a flame ionization detector. Chromatographic conditions followed Sawada et al. (2014): capillary column with a nonbonded poly(biscyanopropyl siloxane) phase (0.20 µm, 100 m × 0.25 mm i.d.) (SP‐2560, Supelco, Bellefonte, PA, USA); helium as a carrier gas (rate of 0.74 mL/min); injection temperature of 250°C; column temperature of 140°C (held for 5 min), increased to 240°C (rate of 4°C/min), held at 240°C for 15 min; a detection temperature of 260°C; and an injection volume of 1.0 µL. The identification of the FAMEs used external standards from Supelco (Merck, Germany), while the quantification was based on the area ratios of each fatty acid to the area of the internal standard, methyl tridecanoate C13:0 from Sigma–Aldrich (Bellefonte, PA, USA), using the response correction factors of the flame ionization detector and the conversion of FAMEs to fatty acids.

Iodine and saponification values were calculated from the fatty acid profile using AOCS methods Cd 1c‐85 and Cd 3a‐94 (2009). TAG composition was estimated from the fatty acid profile using the statistical method of Antoniosi Filho et al. (1995) in MATLAB (MathWorks, USA), assuming a trisaturated TAG content of 80.08% (Sampaio Neto et al. 2020).

Free acidity was determined by titration (IUPAC 2201, 1979) using 0.01 N NaOH in an automatic burette (Metrohm Dosimat 775, Switzerland). Moisture was measured by Karl Fischer titration (AOCS Ca 2e‐84, 2009; Metrohm 787 KF Titrino).

Oil color was assessed as described in Section 2.2.1, chlorophyll was assessed by AOCS Cc 13i‐96, and total carotenoids were assessed by spectrophotometry at 443 nm (Perkin Elmer Lambda 35, USA) using the calibration equation *y* = 0.1772*x* (*R* ^2^ > 0.998) in *n*‐hexane.

Refractive indices at 25°C and 45°C were measured with a portable refractometer (Atago PAL‐RI, Japan) equipped with automatic temperature compensation. The samples were thermally conditioned for 1 h in a thermostatic bath (Marconi, MA184, Brazil). Immediately upon reaching the desired temperature, the refractive index was measured (resolution of 0.0001 in refractive index and 0.1°C in temperature).

The content of soluble carbohydrates in BO.E was estimated by mass balance, taking into account the masses of soluble carbohydrates in BPC and DS.E (Sections 2.2.1 and 2.2.3).

#### 2.2.5 Experimental Determination of Oil Physical Properties

Density and dynamic viscosities were determined using a Stabinger viscometer (Anton Paar, SVM 3000, Austria), whereas surface tension was assessed using a tensiometer (Dataphysics, OCA 15EC, Germany). All physical properties were determined considering the temperature range of 20.0°C–70.0°C (±0.1°C).

#### 2.2.6 Statistical Analysis

Oil extraction trials were performed with two independent replicates, and characterizations of oils and solids were performed in triplicate. For multiple comparisons (BPC, DS.H, and DS.E), Duncan's test was used (SAS, version 9.3, SAS Institute Inc., Cary, NC, USA). The *t*‐test was used to compare BO.H and BO.E (R software, version 4.4.1, Core Team, The R Foundation, Vienna, Austria, 2022). In both cases, a significance level of 5% was considered.

## 3 Results and Discussion

### 3.1 BPC Characterization

BPC was ground to achieve a uniform particle size, resulting in an average particle diameter of 978 ± 19 µm. Figure S3 shows BPC images before (Figure S3a) and after (Figure S3b) grinding, whereas Table S1 shows the particle size distribution of the ground material. The true and apparent densities were 1383.0 ± 0.5 and 734.6 ± 0.4 kg/m^3^, respectively, corresponding to a bed porosity of 46.8 ± 0.03%. The chemical composition and colorimetric parameters are presented in Table 1.

**TABLE 1:** Chemical composition of BPC and defatted solids from hexane (DS.H) and ethanol (DS.E) extractions.

Chemical composition (mass%)		BPC	DS.H	DS.E
Moisture		3.7 ± 0.9 b	6.2 ± 0.3 a	2.7 ± 0.2 b
Ash^a^		4.2 ± 0.1 b	4.9 ± 0.2 a	4.8 ± 0.1 a
Lipids^a^		10.4 ± 0.1 a	0.64 ± 0.04 c	1.95 ± 0.07 b
Proteins^a^		17 ± 1 a	19 ± 1 a	18 ± 1 a
Starch^a^		0.71 ± 0.05 a	na	0.58 ± 0.01 b
Insoluble fibers^a^		53.3 ± 0.6	na	na
Soluble fibers^a^		3.5 ± 0.3	na	na
Soluble carbohydrates^a^, ^b^		7.2 ± 0.7 a	na	6.2 ± 0.3 b
Nonfiber carbohydrates^a^, ^c^		4 ± 3	na	na
Total carbohydrates^a^, ^c^		—	75 ± 1 a	69 ± 2 b
WAC (g water/g sample)		4.4 ± 0.7 a	3.9 ± 0.4 a	4.6 ± 0.1 a
OAC (g oil/g sample)		1.5 ± 0.3 a	1.15 ± 0.01 a	1.26 ± 0.06 a
Color parameters	L*	46.9 ± 0.3 c	65.3 ± 0.1 a	60 ± 1 b
	a*	7.42 ± 0.02 a	5.1 ± 0.1 c	5.6 ± 0.1 b
	b*	7.42 ± 0.02 c	15.4 ± 0.1 b	16.75 ± 0.04 a
	ΔColor^d^	—	18.8 ± 0.2 a	12.8 ± 0.9 b
	°Hue	67.6 ± 0.1 b	71.7 ± 0.1 a	71.5 ± 0.3 a
Images		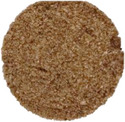	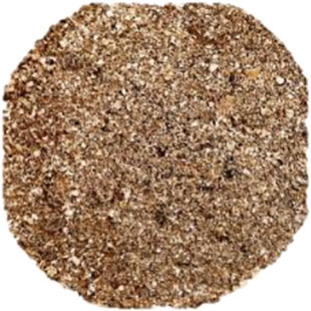	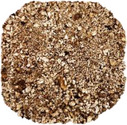

*Note*: The values are means ± standard deviations. The same letters in the same row indicate no significant difference by the Duncan test (*p* > 0.05).

Abbreviation: na, not available.

^a^ Dry basis.

^b^ Soluble carbohydrates expressed as g glucose/100 g of solid (dry basis).

^c^ Calculated by difference considering the sum of lipids, ashes, proteins, starch, and insoluble and soluble fibers.

^d^ Calculated by Equation (3) using BPC as the reference.

The composition of BPC showed slight differences compared to values reported in the literature (Sampaio Neto et al. 2020; Ferreira et al. 2023), likely due to variations in nut processing and plant characteristics. BPC retains residual oil and proteins, supporting strategies for their recovery.

The macro‐ and micronutrient content of BPC includes P (8.2 ± 0.1), K (10.9 ± 0.1), Ca (1.3 ± 0.1), Mg (4.2 ± 0.1), and S (1.88 ± 0.04) (in g/kg), and Cu (36.8 ± 0.4), Fe (64.8 ± 0.3), Mn (440 ± 2), and Zn (56 ± 1) (in mg/kg). These nutrient levels exceed those in Brazil nuts and remain elevated compared with unpressed babassu nuts (Moreda‐Piñeiro et al. 2016; de Oliveira, Mazzali, et al. 2019), highlighting BPC as a particularly rich source of minerals for potential human nutrition.

Aflatoxin levels in BPC were evaluated and found to exceed the 20 µg/kg limit set by ANVISA (2022) for the combined total of B1, B2, G1, and G2 in certain foods. Specifically, B1 was 204.8 ± 0.8 µg/kg, B2 was 3.9 ± 0.4 µg/kg, and G1 was 4.7 ± 0.2 µg/kg, while G2 was not detected. These results indicate that BPC valorization strategies should consider good practices in handling babassu nuts, pressing and storing the cake, and performing solid material decontamination techniques.

**FIGURE 1:**
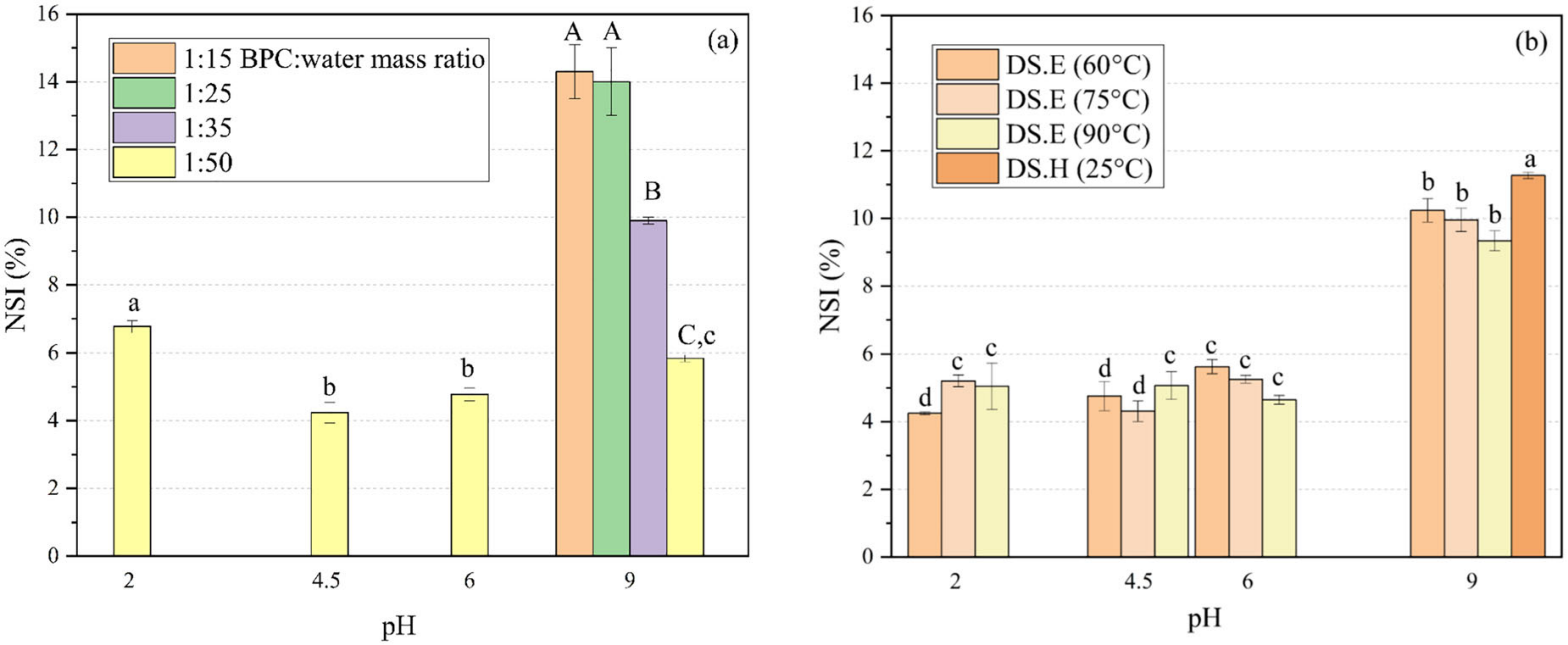
Solubility index (NSI, %) as a function of pH: (a) BPC under different solid‐to‐solvent mass ratios; (b) defatted solids from hexane (DS.H) and ethanol (DS.E) at a solid‐to‐solvent mass ratio of 1:15. The same lowercase letters in the same figure indicate no significant difference for NSI values as a function of pH for the same solid‐to‐solvent mass ratio (Duncan's test, *p* > 0.05). The same uppercase letters in the figure indicate no significant difference for NSI values as a function of solid‐to‐solvent mass ratio for the same pH (Duncan's test, *p* > 0.05).

The solubility of BPC protein in water was assessed across a pH range of 2.0 ± 0.5 to 9.0 ± 0.5 and at BPC‐to‐water mass ratios of 1:15, 1:25, 1:35, and 1:50 at 25.0°C ± 0.1°C (Figure 1a). At the highest dilution (1:50), protein solubility remained low, with only slight increases at extreme pH values (pH 2 and 9; *p* > 0.05). The NSI values were much lower than those reported for other press cakes such as baru (39 ± 1%) (Aracava et al. 2022), macadamia (48 ± 2%) (Navarro and Rodrigues 2018), sesame seed (32.9 ± 0.6%) (Capellini et al. 2019), soybean (∼20%), canola (∼20%), and sunflower (∼25%) (Vidal et al. 2022).

To increase the protein nitrogen content, the NSI was measured at BPC‐to‐water ratios of 1:15, 1:25, and 1:35 under alkaline conditions (pH 9.0 ± 0.5) at 25°C (Figure 1a). The NSI increased as the amount of solvent decreased. This can be explained by solubility equilibrium, whereby an adequate driving force is required to promote protein extraction. At lower ratios (e.g., 1:50), nitrogen becomes more diluted in the medium, resulting in lower concentrations and potentially reducing the analytical method's sensitivity. Ratios of 1:15 and 1:25 produced statistically similar results (*p* > 0.05), indicating that these conditions strike a better balance between solvent availability and protein concentration. This favors the protein–solvent interaction while avoiding excessive dilution effects, resulting in greater detection of solubilized nitrogen. Based on this, the 1:15 ratio was selected for subsequent experiments (Figure 1a).

### 3.2 BPC Residual Oil Recovery Using Solvent Extraction

**FIGURE 2:**
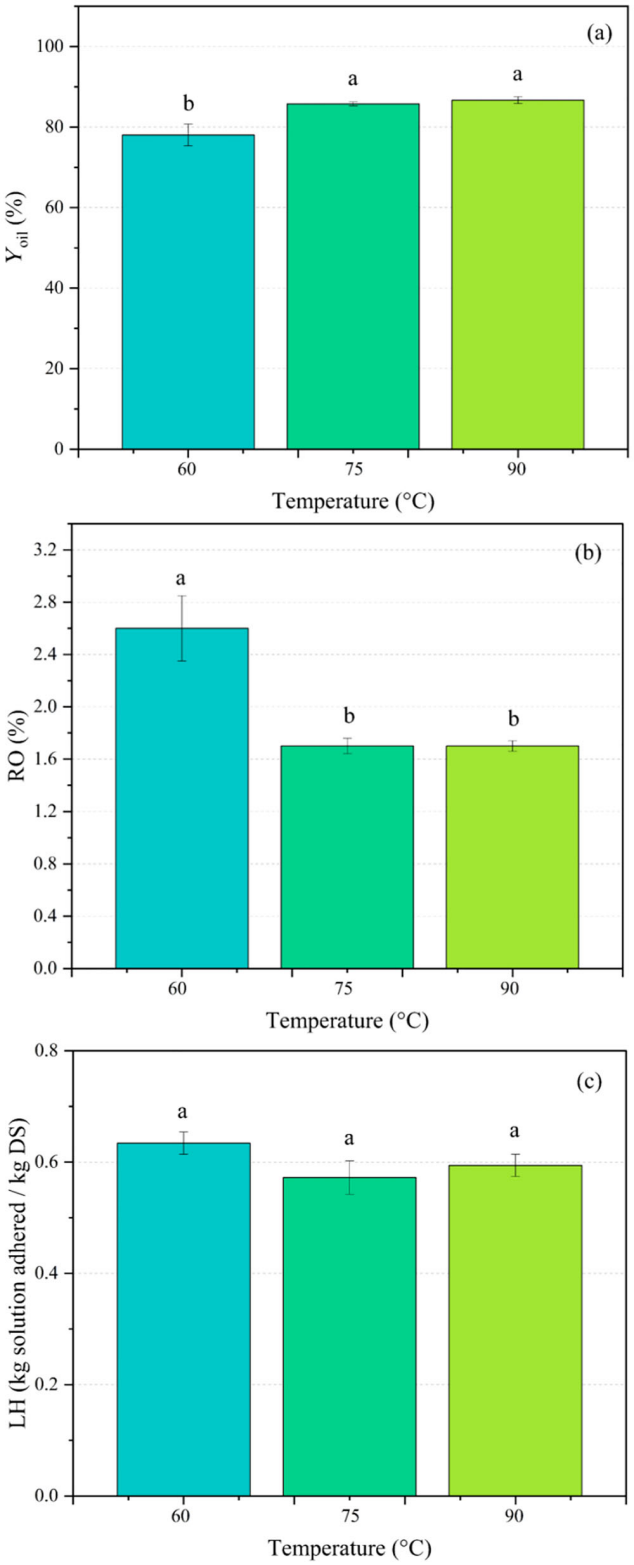
(a) Babassu oil extraction yield (*Y*_oil_, %), (b) residual oil in the defatted solid (RO, %), and (c) liquid holdup (LH, kg solution adhered/kg DS) for the extraction trials at 60°C, 75°C, and 90°C with ethanol. The same lowercase letters in the same row indicate no significant difference (Duncan's test, *p* > 0.05).

Oil extraction from BPC was performed using hexane and ethanol to obtain BO.H and BO.E, along with the corresponding DSs (DS.H and DS.E) for characterization. Hexane extraction was done at 25°C for comparison, while ethanol extraction was conducted in a single stage at 60°C, 75°C, and 90°C (Figure 2).

The results in Figure 2a demonstrate that extracting residual oil from BPC using ethanol is feasible, with yields exceeding 80% under all conditions. Increasing the extraction temperature reduced the residual oil (Figure 2b) in the DS (DS.E), confirming the efficiency of lipid removal. However, no significant differences were observed between 75°C and 90°C (*p* > 0.05). In this study, oil yields were 78 ± 3% at 60°C, 85.8 ± 0.5% at 75°C, and 86.7 ± 0.9% at 90°C (Figure 2a), indicating improved extraction efficiency compared to Sampaio Neto et al. (2020). Those authors performed solid–liquid equilibrium experiments varying the BPC‐to‐solvent mass ratio (1:20–3:5) and temperature (25°C–45°C) for 48 h, obtaining yields of 57%–75% regardless of the ratio or temperature. As detailed in Section 2.2.2, the extractor used to perform the trials is hermetically sealed to prevent solvent loss and equipped with a manometer that registered 0.25 bar at 60°C, 0.50 bar at 75°C, and 0.60 bar at 90°C.

Regarding LH (Figure 2c), no significant differences were observed as temperature increased (*p* > 0.05). These results are consistent with those of Sampaio Neto et al. (2020), who reported about 0.7 kg of adhered solution per kilogram of fiber at 45°C. The LH values for BPC were lower than those reported for other press cakes, such as macadamia (2.7 kg solution/kg fiber) (Navarro and Rodrigues 2018) and peanut (1.6 kg solution/kg fiber) (Magalhães et al. 2023), and similar to pretreated materials like rice bran and corn germ pellets (∼0.4 kg solution/kg fiber) (Toda et al. 2023). As noted by Toda et al. (2023), pretreatments such as pelleting increase solid porosity, improving drainability and percolation. Although BPC was not pelleted, its high insoluble fiber content (∼53%, Table 1) and bed porosity (∼47%), which is greater than that of peanut press cake (∼28%), may have promoted solvent drainage. Low LH values are advantageous in solvent extraction because they indicate less retained solution, meaning less retained oil and solvent. Consequently, less energy is required for solvent recovery.

Based on these results, it is possible to estimate the energy costs of recovering the solvent adhering to the DS per ton of BPC processed. This estimation accounted only for the mass of the DSs obtained from the extraction process (1.34 ton), the amount of extract adhering to the solid phase (38%), and the heat of vaporization of ethanol (854 kJ/kg). Babassu oil extraction using ethanol requires approximately 435 MJ per ton of BPC to recover the solvent in this specific step. If only the differences in vaporization latent heat were considered, the costs of ethanol extraction would be 2.6 times those of hexane extraction. This estimate agrees with a study by Carré et al. (2024) on rapeseed oil extraction, which estimated 3.3 times the total energy for ethanol extraction versus hexane. Despite ethanol's latent heat of vaporization being higher than hexane's (333 kJ/kg), the benefits of using this renewable solvent can mitigate the increased energy consumption.

Previous studies have evaluated oil extraction from press cake with ethanol as a solvent (Aracava et al. 2022; Magalhães et al. 2023; Navarro and Rodrigues 2018; Capellini et al. 2019). In addition to evaluating the oil extraction yield, it is important to assess how the association between alcohol use and extraction temperature affects the nitrogen solubility of the protein fraction present in the DS. Studies using macadamia and peanut press cakes reported higher NSI values for DS from ethanolic extractions at 75°C than those at 60°C and 90°C (Navarro and Rodrigues 2018; Magalhães et al. 2023). Conversely, for sesame seed press cake, the NSI values were negatively affected by ethanol when oil extraction was accomplished at temperatures above 80°C.

Based on these findings, the NSI of the solids defatted with ethanol (DS.E) at 60°C, 75°C, and 90°C was measured at pH 2, 4.5, 6, and 9 (Figure 1b), using a DS‐to‐water mass ratio of 1:15. For comparison, the NSI of the hexane‐defatted solid (DS.H) was also determined and shown in the same figure. According to Figure 1b, the extraction temperature with ethanol (60°C, 75°C, and 90°C) did not significantly affect the NSI of DS.E (*p* > 0.05). At each pH, the values were statistically equivalent (*p* > 0.05), with the highest NSI observed at pH 9. Comparing DS and BPC (Figure 1a) at pH 9 and a 1:15 solid‐to‐solvent ratio shows that lipid extraction reduced protein solubility, regardless of solvent or temperature. Although DS.H exhibited a higher NSI (11.27 ± 0.09%) than DS.E (*p* ≤  .05), all DS samples had lower values than BPC.

Magalhães et al. (2023) reported an NSI of 97% at pH 9 for peanut press cake defatted with ethanol at 75°C, while Aracava et al. (2022) obtained 35% for baru press cake at 60°C. These materials have much lower insoluble fiber levels (3.9 ± 0.1% and 25.2 ± 0.5%, respectively) than BPC (53.3 ± 0.6%; Table 1). According to Schutyser et al. (2015), high crude fiber and water‐insoluble cell wall content promote entanglement between proteins and starch granules, suggesting that fiber acts as a limiting factor for protein solubility.

Based on these results, 75°C was chosen for lipid extraction with ethanol and subsequent oil characterization (Figure 2). Compared to 60°C, this temperature lowers oil and solvent viscosity, improving solubility, diffusion, and, consequently, mass transfer (Bessa et al. 2017). Compared to 90°C, extraction at 75°C produced statistically similar BO extraction yields (Figure 2a) and NSI values (Figure 1b).

### 3.3 Characterization of Oils From Extractions via Hexane and Ethanol

The fatty acid compositions of the babassu oils obtained with hexane at 25°C (BO.H) and with ethanol at 75°C (BO.E) are presented in Table 2, whereas the probable TAG compositions are shown in Table 3.

**TABLE 2:** Fatty acid compositions (mass %) of babassu oils from hexane (BO.H) and ethanol (BO.E) extractions.

Fatty acid	C*x*:*y* ^a^	BO.H	BO.E
Caprylic (Cp)	C8:0	5.9 ± 0.1 a	6.4 ± 0.5 a
Capric (C)	C10:0	5.71 ± 0.08 a	6.0 ± 0.3 a
Lauric (L)	C12:0	45.49 ± 0.07 a	45.8 ± 0.3 a
Myristic (M)	C14:0	14.63 ± 0.04 a	14.0 ± 0.4 b
Palmitic (P)	C16:0	8.31 ± 0.04 a	7.9 ± 0.4 a
Stearic (S)	C18:0	3.74 ± 0.03 a	3.6 ± 0.2 a
Oleic (O)	C18:1	13.27 ± 0.01 a	13.03 ± 0.03 b
Linoleic (Li)	C18:2	2.92 ± 0.01 b	3.3 ± 0.1 a
Average molar mass (g/mol)		213.53	212.51
Unsaturated/saturated mass ratio		0.1932	0.1945
Saturated fatty acids (mass %)		83.81	83.72
Unsaturated fatty acids (mass %)		16.19	16.28
Iodine value (g iodine/100 g oil)		16.47	16.83
Saponification value (mg KOH/g oil)		247.98	249.10
Free acidity (mass %)		0.31 ± 0.03 b	0.681 ± 0.004 a
Refractive index (25°C)		1.4563 ± 0.0001 b	1.448 ± 0.004 a
Refractive index (45°C)		1.4558 ± 0.0003 a	1.4509 ± 0.0001 b
Soluble carbohydrates (mass %)^b^		na	5.8 ± 0.2
Moisture (mass %)		0.22 ± 0.01 a	0.17 ± 0.01 b
Total carotenoids (mg/ g)		nd	nd
Chlorophyll (mg/ kg)		0.05 ± 0.00 b	0.27 ± 0.02 a
Color parameters	L*	90.03 ± 0.02 a	89.93 ± 0.05 b
	a*	−1.97 ± 0.02 a	−3.77 ± 0.03 b
	b*	10.28 ± 0.04 b	17.4 ± 0.5 a
	°Hue	100.85 ± 0.01 b	102.23 ± 0.01 a
Images		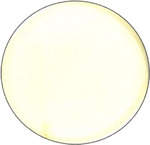	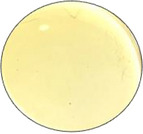

*Note*: The values are means ± standard deviations. The same letters in the same row indicate no significant difference by the *t*‐test (*p* > 0.05).

Abbreviations: na, not available; nd, not detected.

^a^ C*x* = number of carbons and *y* = number of double bonds.

^b^ Calculated by mass balance. The values are means ± standard deviations. The same letters in the same row indicate no significant difference by the *t*‐test (*p* > 0.05).

**TABLE 3:** Probable triacylglycerol composition (mass %) of babassu oils from hexane (BO.H) and ethanol (BO.E) extractions.

	Main group^a^	Triacylglycerol^b^	BO.H	BO.E
TAG composition	28:0	CpCpL	1.07	1.26
	30:0	CpCL	1.95	2.21
	32:0	LLCp	7.22	7.85
	34:0	LLC	9.0	9.41
	36:0	LLL	17.47	17.55
	38:0	LLM	13.89	13.42
	40:0	LLP	10.13	9.53
	42:0	LLS	6.31	5.84
	44:0	PPL	2.82	2.54
	46:0	LPS	1.18	1.05
	38:1	CpLO	0.8	0.85
	40:1	CLO	0.86	0.88
	42:1	LLO	2.58	2.52
	44:1	LMO	1.48	1.38
	46:1	LPO	0.93	0.86
	42:2	LLLi	0.56	0.63
	44:2	OOCp	1.64	1.69
	46:2	OOC	1.31	1.31
	48:2	OOL	7.94	7.55
	50:2	OOM	2.31	2.1
	52:2	OOP	1.2	1.08
	44:3	CpOLi	0.58	0.68
	46:3	CLiO	—	0.55
	48:3	LOLi	3.43	3.73
	50:3	MOLi	1.0	1.04
	52:3	POLi	0.52	0.54
	54:3	OOO	1.16	1.1
	54:4	OOLi	0.64	0.68

^a^ *x*:*y*, *x* = number of carbons and *y* = number of double bonds, without considering glycerol.

^b^ Groups with total triacylglycerol composition lower than 0.5% were ignored. The values are means ± standard deviations.

Table 2 shows that babassu oils contain high levels of lauric and myristic acids, regardless of the extraction solvent. The high concentration of saturated fatty acids (around 83% of the total composition) results in oils with low iodine values and high saponification indices. These results are consistent with those reported in the literature. No significant differences in the fatty acid composition of babassu oil were observed when comparing oil obtained by pressing nuts (Bauer et al. 2020; Codex Alimentarius Commission 1999) with oil obtained by solvent extraction with hexane (BO.H) or ethanol (BO.E) from BPC. BO.H and BO.E were also compared with babassu oils obtained by supercritical CO_2_ extraction from nuts (de Oliveira, Mazzali, et al. 2019), pressurized liquid ethanol and isopropanol extraction from nuts (de Oliveira, dos Santos Garcia, et al. 2019), and ethanolic extraction from BPC (Sampaio Neto et al. 2020). The extraction method does not affect the fatty acid or TAG composition, suggesting that a renewable solvent could be used to recover residual babassu oil from the press cake.

Free acidity, an indicator of oil quality and hydrolytic degradation, was expressed as lauric acid. BO.H complied with the Codex Alimentarius (1999) limit of 0.3%. The higher acidity observed for BO.E is consistent with findings by Magalhães et al. (2023), who reported that ethanol extraction was associated with increased free fatty acid content due to elevated temperatures that promote TAG hydrolysis. Moreover, ethanol facilitates alcoholysis reactions, intensifying acyl exchange and thereby increasing oil acidity (Li et al. 2014).

The content of soluble carbohydrates in BO.E is shown in Table 2 and was estimated by mass balance based on the contents of soluble carbohydrates in BPC and DS.E (Table 1). The high content of soluble carbohydrates in BO.E corroborates the extraction of polar compounds by ethanol.

Babassu oils obtained by both extraction methods showed high luminosity. The a* parameter had negative values, suggesting a greater intensity of the green hue, which was most likely associated with the chlorophyll content. The b* parameter indicated a predominance of yellow, which was more intense in the ethanol‐extracted oil (BO.E) (see images in Table 2), regardless of the lack of detection of carotenoids in the oils.

These findings emphasize the need to refine both hexane‐ and ethanol‐extracted oils, particularly BO.E, due to its higher free acidity and more intense color.

The moisture content of BO.E was lower than that of BO.H, likely due to the drying method employed. While BO.H was dried in a fume hood to constant mass, BO.E underwent vacuum drying, which promotes more efficient moisture removal.

The refractive indices of the oils at 25°C and 45°C did not differ significantly, indicating that the solvent type did not affect this parameter. These findings align with values reported for babassu oil obtained by nut pressing (1.448–1.451 and 1.449 at 40°C) (Codex Alimentarius Commission 1999; Bauer et al. 2020) and by supercritical CO_2_ extraction (1.4560 at 25°C and 1.4520 at 45°C) (de Oliveira, Mazzali, et al. 2019).

**FIGURE 3:**
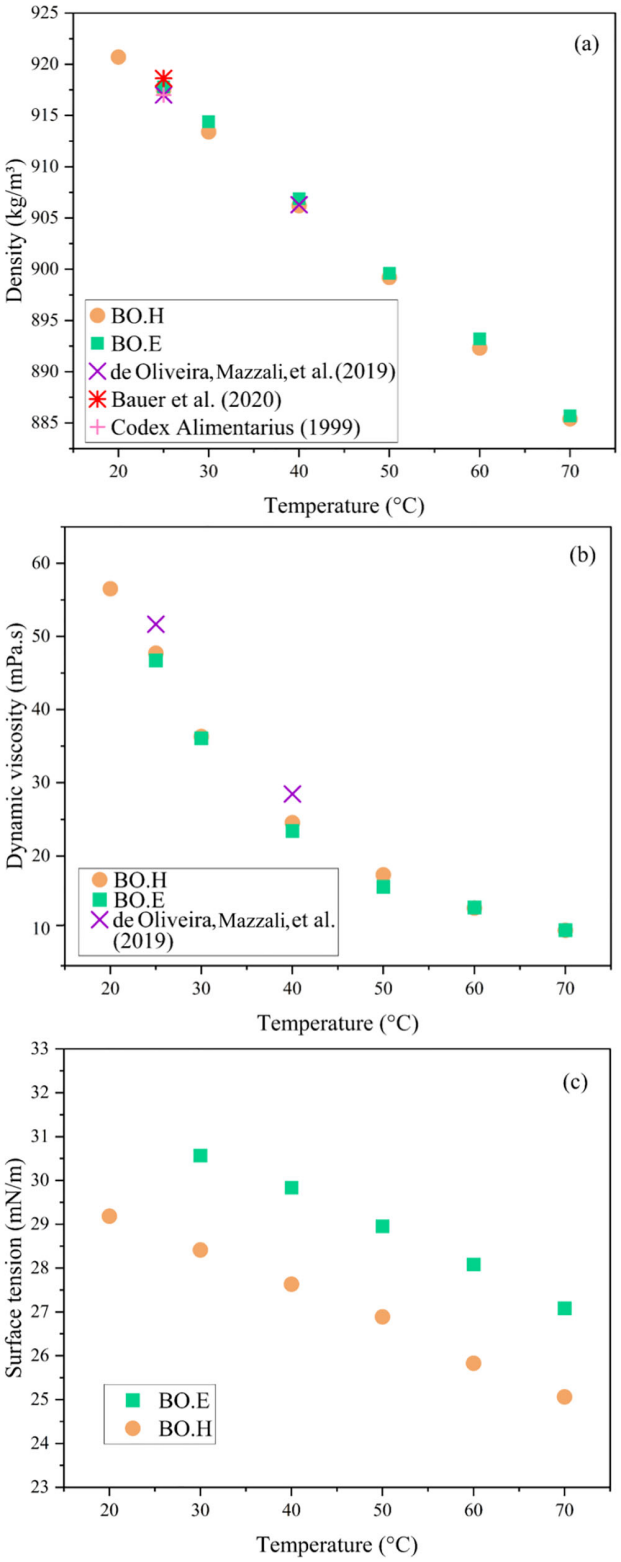
Physical properties of oils extracted from BPC with hexane (BO.H) and ethanol (BO.E) as a function of temperature: (a) density, (b) dynamic viscosity, and (c) surface tension.

The physical properties of BO.H and BO.E, including density, dynamic viscosity, and surface tension, are shown in Figure 3. As expected, these properties decreased with increasing temperature. The density (Figure 3a) and viscosity (Figure 3b) values were similar for both oils. The results are consistent with literature data for babassu oils obtained either by nut pressing (Codex Alimentarius 1999; Bauer et al. 2020) or by supercritical CO_2_ extraction (de Oliveira, Mazzali, et al. 2019).

These are the first reported surface tension data for babassu oils (Figure 3c). Higher values were observed for BO.E than for BO.H. This property is highly sensitive to minor contaminants, and although both oils were desolventized, residual solvent traces may still influence the results. The surface tensions of hexane and ethanol were determined at 30°C, yielding values of 16.94 ± 0.06 and 21.64 ± 0.05 mN/m, respectively. This suggests that the higher BO.E surface tension values may be due to ethanol residues.

The use of ethanol for recovering residual lipids from BPC demonstrated high efficiency, yielding an oil with characteristics similar to those of oils extracted with the fossil solvent and to those of pressed nuts. These results reveal the potential of incorporating residual oil from BPC in various applications, increasing the oil supply on the market, and contributing to the valorization of the entire babassu production chain.

### 3.4 Characterization of Defatted Solids From Oil Extractions via Hexane and Ethanol

Table 1 also presents the chemical composition and color parameters of DSs obtained with hexane at 25°C (DS.H) and ethanol at 75°C (DS.E). DS.E exhibited lower moisture content than DS.H, likely due to the drying methods employed: DS.E was dried in a forced‐convection oven at 50°C for 48 h, and DS.H was dried in a fume hood until constant mass. Conversely, DS.H contained less residual lipid than DS.E, which can be attributed to the three‐stage extraction and to hexane's higher lipid solubilization capacity, even at room temperature. Protein, carbohydrate, and ash contents were not significantly different between treatments (*p* > 0.05).

The solid defatted with ethanol presented a total aflatoxin B1 content of 165 ± 1 µg/kg, a value substantially higher than that permitted by legislation (ANVISA 2022) but lower than that determined in the BPC. Aflatoxin B2 and G1 levels in the DS.E were 3.91 ± 0.07 and 4.6 ± 0.3 µg/kg, respectively, similar to those measured in the BPC. Aflatoxin G2 was not detected in any of the samples. Although the single‐stage ethanol‐based oil extraction step was not fully effective at decontaminating BPC, the results indicate that the renewable solvent can partially reduce aflatoxin levels. However, the reduction was partial, and the material is still unsafe for direct food use without further decontamination.

The color parameters of both DSs differed from BPC. Significant differences were observed in the L*, a*, and b* values, with DS.E exhibiting a more intense yellow hue than DS.H. The lower a* values of the DSs compared to the BPC (see Table 1) can be attributed to the leaching of pigments, particularly chlorophyll, from the BPC into the oils (see Table 2). This indicates that pigments were extracted by the solvents during lipid recovery. The total color change (ΔColor), calculated relative to BPC, was higher in DS.H than in DS.E, likely due to the lower residual oil content in DS.H. These results suggest that, in addition to recovering residual lipids, solvent extraction removes pigments, producing a DS that may serve as a valuable source of fibers and proteins.

Table 1 shows similar WAC and OAC values for BPC, DS.E, and DS.H, indicating that the extraction of residual lipids from BPC did not significantly affect these properties, with WAC values not statistically different. Moreover, for OAC, it was lower for DS.H, which also had a lower lipid content than BPC and DS.E. Aracava et al. (2022) reported values of 2.30 ± 0.04 g water/g sample and 0.95 ± 0.03 g oil/g sample for baru nut cake. For the DS from the ethanolic extractions at 60°C, the authors determined 3.10 ± 0.02 g water/g sample and 1.18 ± 0.03 g oil/g sample. The greatest improvements in WAC and OAC with oil extraction are observed for the macadamia press cake. Navarro and Rodrigues (2018) reported values of 1.8 ± 0.1 g water/g sample and 1.4 ± 0.1 g oil/g sample for the press cake and higher values for the DS (5.0 ± 0.2 g water/g sample and 3.5 ± 0.3 g oil/g sample).

High WAC values are desirable in viscous foods, such as sausages and baked goods, as they promote water retention without inducing protein dissociation, thereby enhancing texture and thickening properties. However, excessive WAC may lead to dehydration of other formulation components (Garcia‐Vaquero et al. 2017). Similarly, high OAC values are advantageous, enabling the use of plant‐based proteins as fat replacers in meat analogs, mayonnaise, salad dressings, and other applications (Capellini et al. 2020; Garcia‐Vaquero et al. 2017).

## 4 Conclusions

The chemical characterization of babassu nut press cake (BPC) revealed a material rich in insoluble fibers, residual oil, protein, and macro‐ and microminerals. BPC stands out for its content of phosphorus, potassium, magnesium, copper, iron, manganese, and zinc, highlighting its potential for human nutrition.

The use of a renewable solvent, ethanol, for residual lipid recovery proved efficient, achieving high oil extraction yields with low LH. Conversely, the combination of high insoluble fiber content and low NSI values indicates that protein recovery from BPC remains challenging.

Babassu oil obtained with ethanol exhibited fatty acid and TAG compositions and physical properties similar to oils extracted with hexane or directly from nut pressing. Beyond oil recovery, solvent extraction produced a DS rich in fibers and proteins, with water and oil absorption capacities suitable for food applications. Ethanol extraction also reduced aflatoxin levels, particularly aflatoxin B1. However, the reduction was partial, and the material is still unsafe for direct food use without further decontamination.

These findings demonstrate the potential to incorporate residual babassu oil into various applications, increasing market supply, while the use of defatted BPC in food formulations contributes to the valorization of the entire babassu production chain.

## Author Contributions

**Clara Santa Rosa Fioriti**: conceptualization, data curation, formal analysis, investigation, methodology, software, writing – original draft. **Paloma Jamily Cristina Magalhães**: methodology, formal analysis. **Paola de Cássia Franco Visioli**: formal analysis, methodology. **Keila Kazue Aracava**: methodology, investigation. **José Pedro Zanetti Prado**: formal analysis, investigation. **Christianne Elisabete da Costa Rodrigues**: conceptualization, funding acquisition, methodology, project administration, resources, supervision, validation, visualization, writing – review and editing.

## Conflicts of Interest

The authors declare no conflicts of interest.

## Supporting information



Supplementary data associated with this article can be found online. This document presents an experimental setup (Figure S1), a scheme of sequential extraction in cross‐current configuration (Figure S2), pictures of babassu press cake (BPC) as received and after grinding (Figure S3), and the particle size distribution of babassu press cake (Table S1).

## Data Availability

The data presented in this study are available on request from the corresponding author.
