# Supplementary material for: How Does the Extraction of Remaining Lipids From Babassu Press Cake Using Green and Traditional Solvents Affect the Oil and Defatted Solids?
Source: J Food Sci. 2026 Jul 7;91(7):e71275. doi: 10.1111/1750-3841.71275 (PMC13342453; doi:10.1111/1750-3841.71275)
Supplement: Supplementary file 1 — Supplementary data associated with this article can be found online. This document presents an experimental setup (Figure S1), a scheme of sequential extraction in cross‐current configuration (Figure S2), pictures of babassu press cake (BPC) as received and after grinding (Figure S3), and the particle size distribution of babassu press cake (Table S1). [file JFDS-91-0-s001.docx]

**How does the extraction of remaining lipids from babassu press cake using green and traditional solvents affect the oil and defatted solids?**

Clara Santa Rosa Fioriti^a^, Paloma Jamily Cristina Magalhães^a^, Paola de Cássia Franco Visioli^a^, Keila Kazue Aracava^a^, Ingrid Denardi Soares^a^, José Pedro Zanetti Prado^a^, Christianne Elisabete da Costa Rodrigues^a,^*

^a^ Laboratório de Engenharia de Separações (LES), Departamento de Engenharia de Alimentos (ZEA), Faculdade de Zootecnia e Engenharia de Alimentos (FZEA), Universidade de São Paulo (USP), P.O. Box 23, 13635-900 Pirassununga, São Paulo, Brazil.

*Corresponding author.

chrisrodrigues@usp.br (Christianne E. C. Rodrigues, Ph.D. Professor).

***Supplementary material***


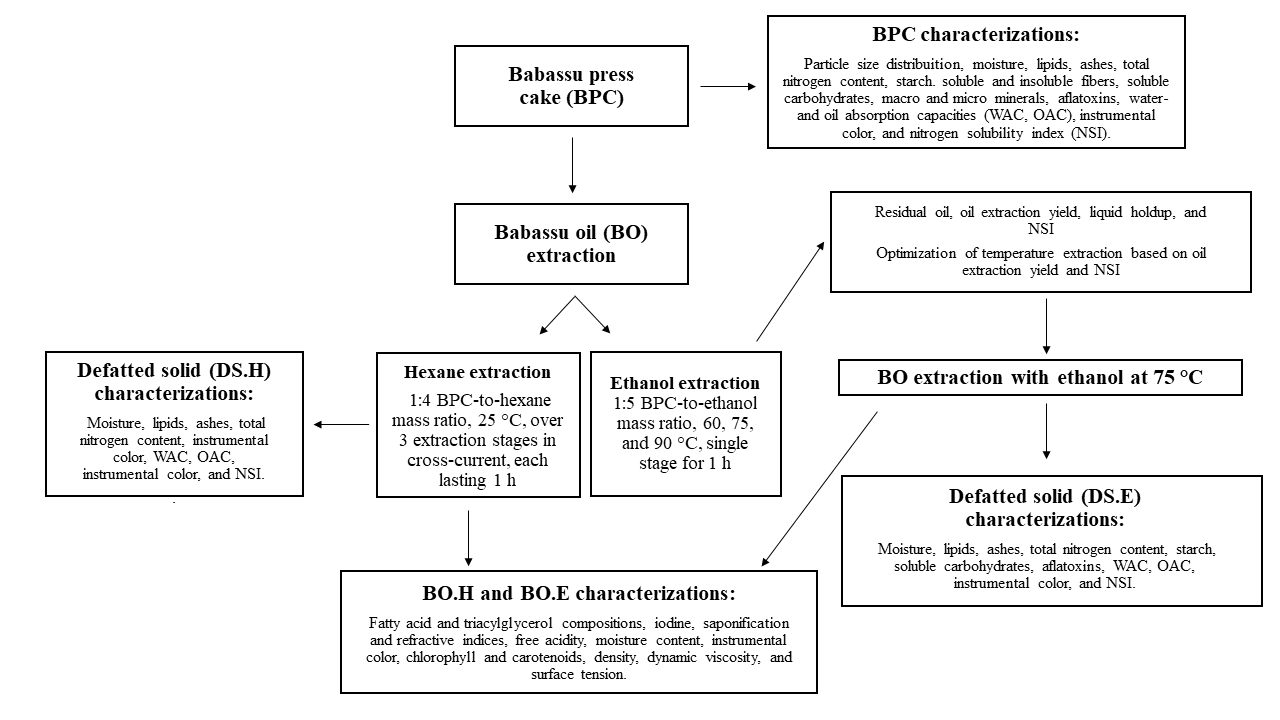


**Figure S1.** Experimental setup.


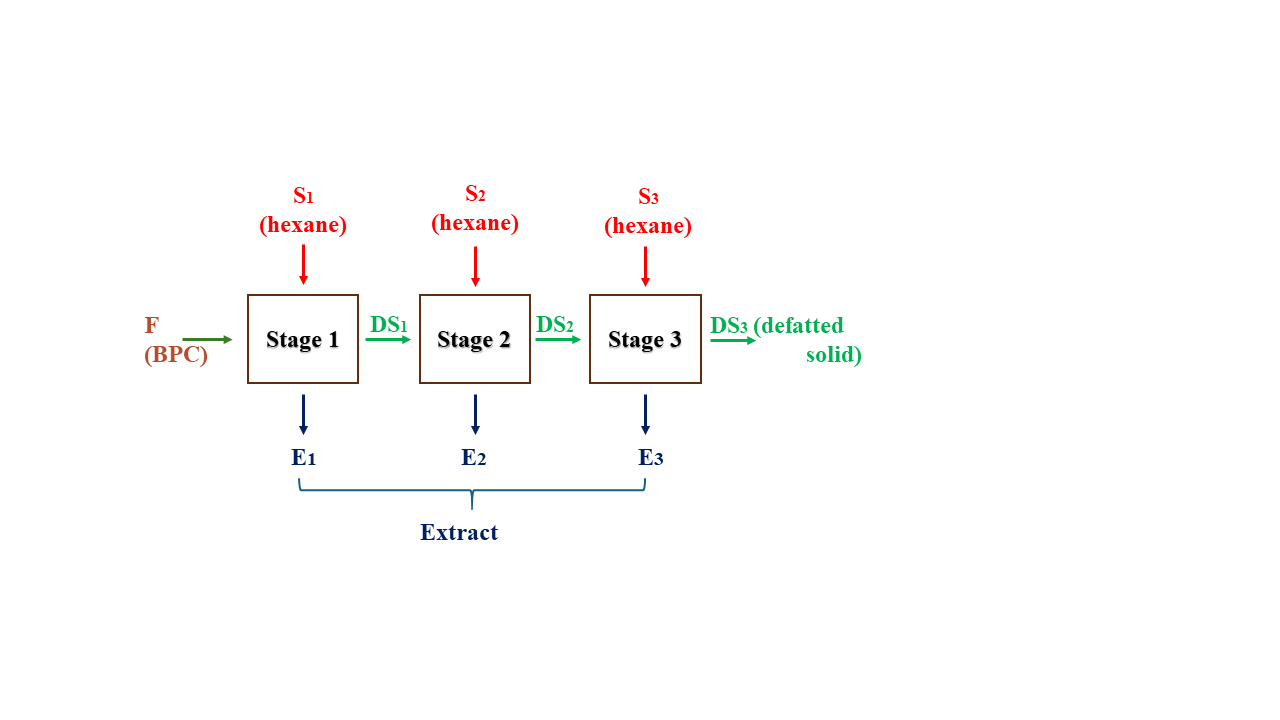


**Figure S2.** Sequential extraction in cross current configuration.

| 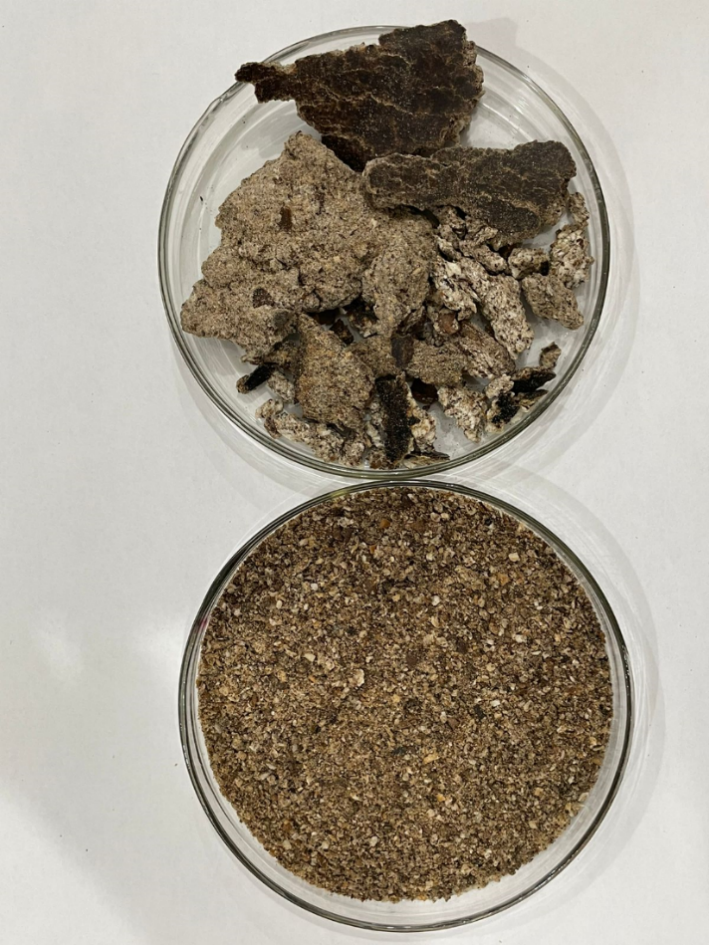 | 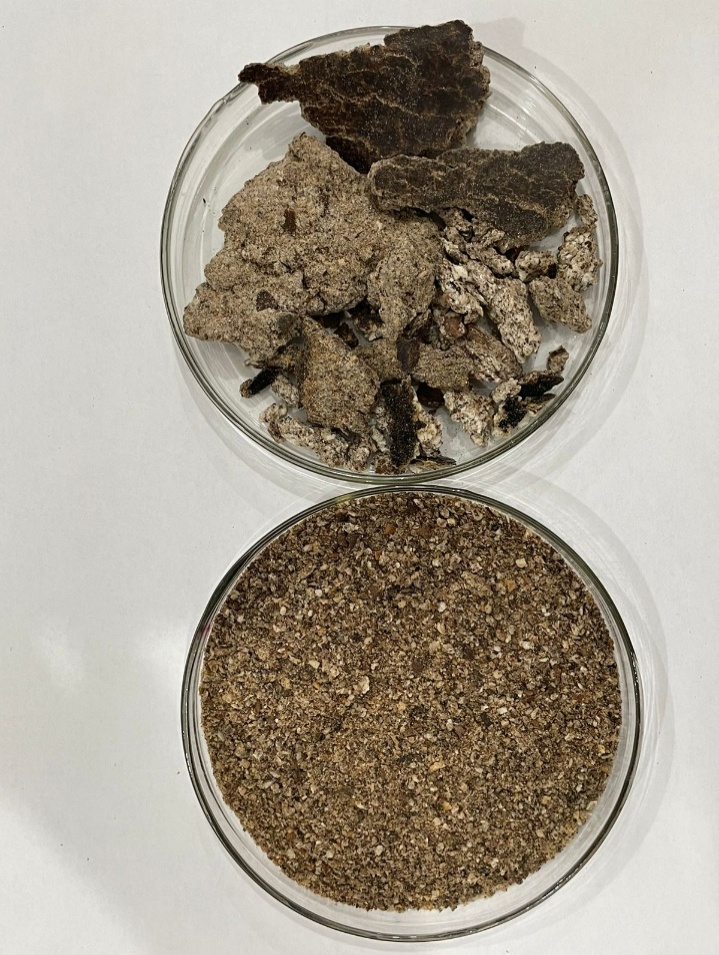 |
| --- | --- |
| (a) | (b) |

**Figure S3.** Babassu press cake (BPC) as received (a) and after grinding (b).

**Table S1**. Particle size distribution of babassu press cake (BPC).

| Mesh | di (µm) | di+1 (µm) | BPC mass (g) | % | Average  particle size (µm) |
| --- | --- | --- | --- | --- | --- |
| 8 | 2360 | 3350 | 0.25 ± 0.06 | 1.0 ± 0.3 | 978 ± 19 |
| 9 | 2000 | 2360 | 1.5 ± 0.1 | 6.0 ± 0.4 |  |
| 10 | 1680 | 2280 | 2.6 ± 0.2 | 10.4 ± 0.7 |  |
| 14 | 1190 | 1680 | 6.9 ± 0.3 | 27.3 ± 1 |  |
| 20 | 841 | 1190 | 5.2 ± 0.2 | 20.6 ± 0.6 |  |
| 28 | 595 | 841 | 2.48 ± 0.04 | 9.9 ± 0.1 |  |
| 35 | 420 | 595 | 3.8 ± 0.7 | 14.9 ± 2.8 |  |
| 48 | 297 | 420 | 2.3 ± 0.8 | 9 ± 3 |  |
| Bottom pan | 7 | 297 | 0.03 ± 0.02 | 0.12 ± 0.09 |  |
